# Hypoglycemic Effect of Electroacupuncture at ST25 Through Neural Regulation of the Pancreatic Intrinsic Nervous System

**DOI:** 10.1007/s12035-021-02609-1

**Published:** 2021-11-10

**Authors:** Tiancheng Xu, Zhi Yu, Yun Liu, Mengjiang Lu, Meirong Gong, Qian Li, Youbing Xia, Bin Xu

**Affiliations:** 1 Key Laboratory of Acupuncture and Medicine Research of Ministry of Education, Nanjing University of Chinese Medicine, Nanjing, 210023 China; 2 Affiliated Hospital of Xuzhou Medical University, Xuzhou Medical University, Xuzhou, 221004 China

**Keywords:** Diabetes, Insulin, TRPV1 receptor, Streptozotocin, Acupuncture, Inflammation

## Abstract

Electroacupuncture (EA) is considered to have potential antidiabetic effects; however, the role of the pancreatic intrinsic nervous system (PINS) in EA-induced amelioration of type 2 diabetes (T2DM) remains unclear. Therefore, we investigated whether EA at ST25 exerts any beneficial effects on insulin resistance (IR), inflammation severity, and pancreatic *β* cell function via the PINS in a rat model of a high-fat diet-streptozotocin (HFD/STZ)-induced diabetes. To this end, Sprague Dawley rats were fed with HFD to induce IR, followed by STZ (35 mg/kg, i.p.) injection to establish the T2DM model. After hyperglycemia was confirmed as fasting glucose level > 16.7 mmol/L, the rats were treated with EA (2 mA, 2/15 Hz) for the next 28 days. Model rats showed increased serum glucose, insulin, IR, and TNF-*α* levels with a concomitant decrease in *β* cell function. Microscopy examination of the pancreas revealed pathological changes in islets, which reverted to near-normal levels after EA at ST25. EA improved islet cell morphology by increasing islet area and reducing vacuolation. EA at ST25 decreased transient receptor potential vanilloid 1 (TRPV1) and increased substance P (SP) and calcitonin gene-related peptide (CGRP) expression. Subsequently, insulin secretion decreased and impaired pancreatic endocrine function was restored through the TRPV1 channel (SP/CGRP)-insulin circuit. EA increased choline acetyltransferase and neuropeptide Y expression and controlled inflammation. It also enhanced the cocaine and amphetamine-regulated transcript prepropeptide expression and promoted glucagon-like peptide-1 secretion. Additionally, the electrophysiological activity of PINS during acupuncture (2.71 ± 1.72 Hz) was significantly increased compared to the pre-acupuncture frequency (0.32 ± 0.37 Hz, *P* < 0.05). Thus, our study demonstrated the beneficial effect of EA on *β* cell dysfunction via the PINS in rat models of HFD-STZ-induced T2DM.

## Introduction

The prevalence of type 2 diabetes mellitus (T2DM) has been rapidly increasing worldwide. According to the latest reports, the total number of patients with diabetes in mainland China is estimated to be 129.8 million [1]. T2DM is strongly associated with obesity [2] and characterized by progressive pancreatic *β* cell dysfunction [3], accompanied by insulin resistance (IR) [4]*.* IR contributes to impaired glucose homeostasis and type 2 diabetes [5, 6]. Pancreatic *β* cell dysfunction is central to the pathogenesis of type 2 diabetes [7]. Preserving *β* cell function during the development of obesity and IR would limit the development of type 2 diabetes [8]. Meanwhile, being overweight increases the risk of metabolic disease; more than a third of the Chinese population has prediabetes [9]. Recent studies have demonstrated that acupuncture could regulate lipid metabolism disorder [10] and improve glucose tolerance (IGT) [11], which is beneficial to the prevention and treatment of T2DM. Acupuncture is effective in the management of various metabolic disorders such as hyperglycemia and overweight by alteration of the sympathetic nervous system and insulin signal defects [12]. Acupuncture has been practiced in East Asian countries to relieve a variety of illnesses and is now widely accepted worldwide [13]. It can facilitate weight control by regulating the nervous, endocrine, and digestive systems [14]. It might also be useful in reducing blood glucose levels in patients with T2DM [15] and improving insulin sensitivity [16]. When combined with metformin, the treatment, including electroacupuncture (EA) at ST25, can be used as an insulin sensitizer to effectively manage the risk of T2DM and obesity [17]. While recent studies confirmed that EA alone can also attenuate blood glucose. EA could effectively ameliorate adipose accumulation of obese men [18]. Stimulating bilateral ST25 could effectively regulate fasting blood glucose, insulin, and lipid metabolism [19]. EA might be an alternative for managing islet function and treating T2DM. However, the mechanisms by which acupuncture regulates islet functions remain to be elucidated.

A potential mechanism for the hypoglycemic effect of acupuncture is a neuroendocrine pathway involving crosstalk among the endocrine, nervous, and immune systems. EA modulates distinct sympathetic pathways. Acupuncture at body surface points can mediate the activities of a variety of somatosensory, autonomic, and target organ reflex pathways. Therefore, EA may influence changes in systemic metabolism. We have previously confirmed that EA at ST25 can regulate the activity of glucose-inhibited neurons and improve lipid metabolism disorders [20]. However, the local related neurological changes of the pancreas and the onset mechanism of EA in T2DM are still unclear. Recent work suggests that the pancreatic intrinsic nervous system (PINS) is involved in glucose homeostasis, insulin sensitivity, and pancreatic *β* cell function, and thus the pathogenesis of diabetes [21]. It suggested that the intrinsic nervous system is one of the neural mechanisms of acupuncture regulating blood glucose.

Intrapancreatic ganglia constitute a complex information-processing center that contains various neurotransmitters and forms an endogenous neural network, which has a major influence on pancreatic endocrine function; those neurotransmitters including choline acetyltransferase (ChAT) and neuropeptide Y (NPY) control inflammatory status, cocaine, and amphetamine-regulated transcript (CART-PT) to promote the secretion of glucagon-like peptide-1 (GLP-1) and a promising axis in TRPV1 channel (SP/CGRP)-insulin circuit [22].

EA can control the inflammatory status and have a positive significance for the management of T2DM. EA at ST25 reduced patients’ inflammation levels, thereby improving insulin sensitivity [23]. While the spinal sympathetic axis evoked by 3 mA EA at ST25 can suppress splenic inflammation [24]. The vagus nerve splits in the celiac ganglion, giving rise to the postganglionic splenic nerve that terminates in the spleen [25]. The spatial resolution of the sensations that can be elicited from the viscera is relatively vague and can be fully explained by the segmental width of the afferent inflow from each viscus. Similar neuromeric segments are also present in the pancreas [26, 27]. ST25 is innervated by T10 [28], whose ganglion segment partially overlaps with the pancreas (innervated by T5-T11 [29, 30]). EA at ST25 can reduce pancreatic inflammation via restraint inflammatory factor and NK-*κ*B [31]. The balance of pro- and anti-inflammatory factors in pancreas can be ameliorated by EA at ST25 [32]. Consequently, we hypothesized that EA at ST25 will lead to a hypoglycemic effect through neural regulation of pancreatic endocrine secretion. We tested this hypothesis in the study presented here.

## Materials and Methods

### Establishment of the Experimental Animal Model

**Fig. 1:**
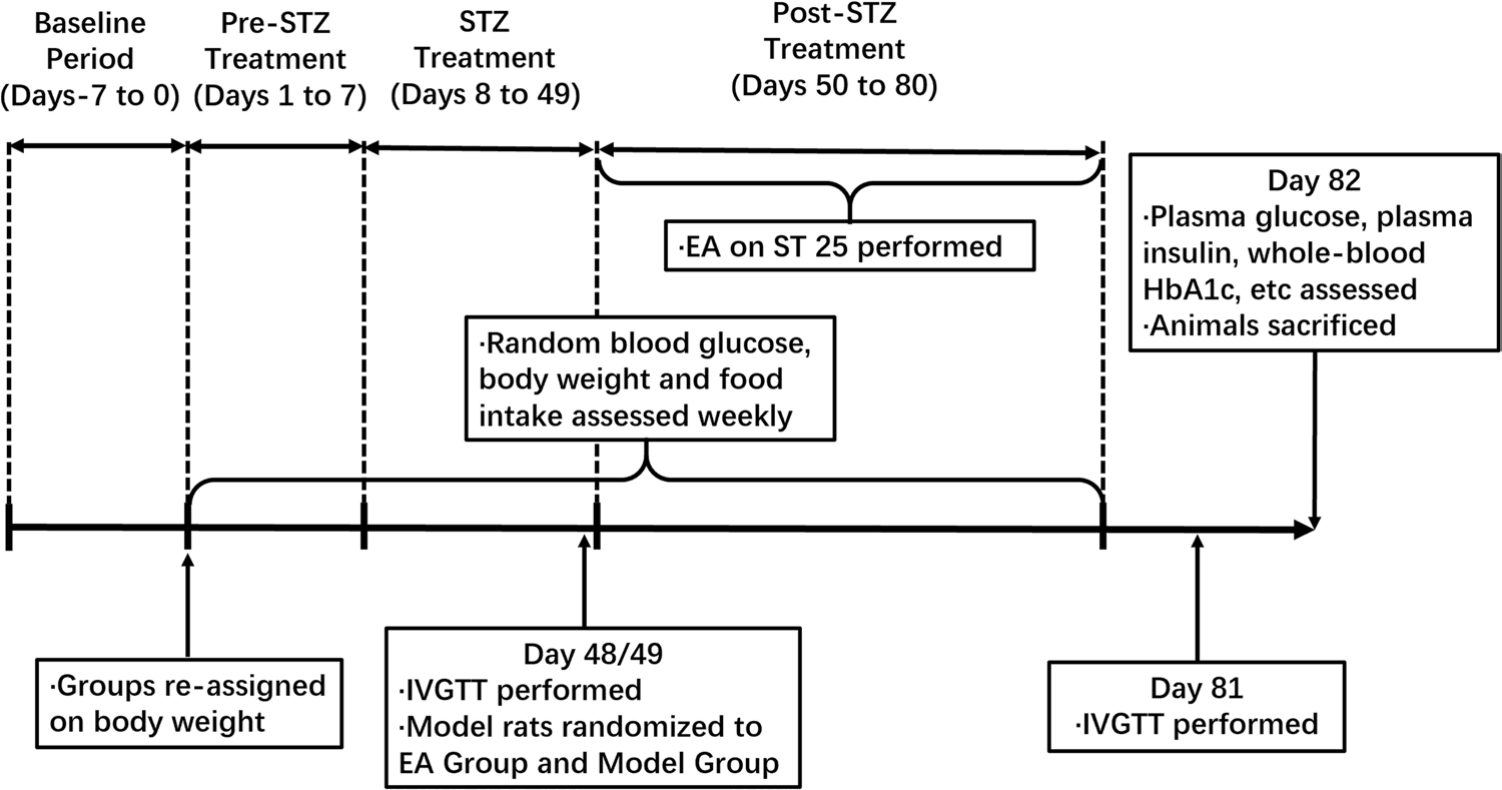
Schedule of the experimental procedures. EA, electroacupuncture; ST25, acupoint Tianshu; STZ, streptozotocin; HbA1c, glycated hemoglobin; IVGTT, intravenous glucose tolerance test

The use of a combination of a high-fat diet (HFD) and a low dose of streptozotocin (STZ) has been shown to effectively establish a rat model of diabetes that mimics the metabolic characteristics of common T2DM in humans [33]. Seven-week-old Sprague Dawley (SD) rats were supplied by the model animal research center of the Nanjing University of Chinese Medicine (No. 1100112011052760, under grant SCXK(JING)2016-0006). The experimental rats were maintained in a controlled environment (conditions: 12-/12-h ± 1-h light/dark cycle; temperature, 22 ± 2 °C; relative humidity 60% ± 5%). The animals were raised in individual cages with ad libitum access to food and water and randomly numbered. They were divided into three groups: the model, EA, and control groups, with six animals each. T2DM was induced by administering a high-fat diet and low-dose STZ (HFD-STZ). The model and EA groups were fasted for 16 h and intraperitoneally (i.p.) injected with STZ (Sigma-Aldrich, St. Louis, MO, USA) dissolved in freshly prepared citrate buffer (0.1 mol/L, pH 4.2) at a dose of 35 mg/kg. Random blood glucose levels were measured 48 h after STZ injection. Rats with a random blood glucose level of >16.7 mmol/L and kept for 2 weeks were considered rats with T2DM. Weight, food intake, and random blood glucose levels were recorded weekly. Random blood glucose levels were monitored weekly by collecting blood from the tail vein and analyzing it with a glucometer (Roche Diagnostics, Mannheim, Germany). To induce IR, the model and EA groups were placed on an HFD [34] consisting of 58% fat, 25% protein, and 17% carbohydrate, as a percentage of total kcal upon arrival, which was maintained for the duration of the study. The control group was fed a standard normal chow diet. The remaining non-T2DM rats in the model and EA groups were killed by cervical dislocation. The schedule of the experimental procedures is shown in Fig. 1. All the experiments were performed per the Principles of Laboratory Animal Care and the Guide for the Care and Use of Laboratory Animals published by the National Science Council, China (under grant 202006A016).

### Blood and Tissue Sample Collection

At the end of the experiment (week 12), the rats fasted for 12 h and anesthetized with isoflurane. Blood samples were drawn from the orbital sinus and centrifuged at 3000 rpm for 15 min at 4 °C. The separated serum was stored at −80 °C for further procedures. The pancreas and duodenum were quickly removed, rinsed, and stored at −80 °C or fixed in 10% paraformaldehyde solution. The metabolic metrics including fasting serum insulin, hemoglobin A1c, leptin, and GLP-1 levels and those of the pro-inflammatory cytokines including tumor necrosis factor-alpha (TNF-α), interleukin 6 (IL-6), and IL-1*β*, and anti-inflammatory cytokines such as interleukin 10 (IL-10) in the serum were determined using rat ELISA kits (Nanjing Jiancheng Bioengineering Institute Co., Ltd.) according to the manufacturer’s instructions.

### Antibodies

Membranes were blocked and probed with primary and secondary antibodies according to the manufacturers’ suggested concentrations. The primary antibodies used are listed in Table 1. The secondary antibodies used were anti-rabbit IgG, HRP-linked antibody (1:2000, Cell Signaling Technology), and anti-mouse IgG, HRP-linked antibody (1:2000, Cell Signaling Technology).

**Table 1:** Primary antibodies used and their respective concentrations

Antibody	Species	Dilution	Source
Transient receptor potential vanilloid 1 (TRPV1)	Mouse	1:1000	Abcam
Choline acetyltransferase (ChAT)	Rabbit	1:1000	Abcam
PGP9.5	Rabbit	1:1000	Abcam
Calcitonin gene-related peptide (CGRP)	Rabbit	1:1000	Abcam
Neuropeptide Y (NPY)	Mouse	1:1000	Santa Cruz
Substance P(SP)	Rabbit	1:1000	Affinity Biosciences
Cocaine and amphetamine-regulated transcript (CART-PT)	Rabbit	1:2000	Signalway Antibody
GAPDH	Rabbit	1:10,000	Cell Signaling Technology
Vinculin	Rabbit	1:2000	Abcam

### Measurement of Random Blood Glucose Levels

Random blood glucose levels were recorded weekly using an Accu-Chek glucometer (Roche Diagnostics, Mannheim, Germany). Animals were free to eat and drink. Homoeostasis model assessment (HOMA) was used to estimate basal *β* cell function (HOMA-*β*) and insulin resistance (HOMA-IR). HOMA-*β* was calculated as follows: 20 × fasting insulin (FINS)/fasting plasma glucose (FPG)—3.5. HOMA-IR was calculated as FPG × FINS/22.5 [35].

### Intravenous Glucose Tolerance Test

Acute insulin secretory response was measured by performing the intravenous glucose tolerance test (IVGTT). Overnight, fasted rats from all the groups were subjected to an oral glucose tolerance test in the last week of the experimental period. The blood glucose levels were monitored at 0, 30, 60, 90, and 120 min by using an Accu-Chek glucometer (Roche Diagnostics, Mannheim, Germany) after intraperitoneal administration of 2 g/kg b.w./rat glucose as an aqueous solution [36].

### Hematoxylin and Eosin Staining

Hematoxylin and eosin (HE) staining was performed according to standard histological protocols. After being carefully isolated, the pancreas was fixed in 4% paraformaldehyde and embedded in paraffin wax. Then, 8-μm-thick sections were obtained with a rotary slicer (Leica, Germany) and mounted on slides. HE staining was performed under a light microscope (Olympus, Japan) to visualize pathological changes.

### Western Blot Analysis

Pancreas tissue (200 mg in weight) was obtained from the animals under anesthesia (0.8 g/kg urethane, i.p.) after 12 h of fasting. The tissue (100 mg) was placed in 1 mL of lysis buffer consisting of protease inhibitor and RIPA buffer (Thermo Fisher Scientific), homogenized, and centrifuged at 14,000 rpm for 30 min. After that, protein concentrations were measured with the BCA Protein Assay Kit (Thermo Fisher Scientific). Then, 20 μg of protein from each sample was resolved electrophoretically on sodium dodecyl sulfate-polyacrylamide gel (12% of separation gel and 5% of concentration gel). Electrophoresis was performed at 70 V for 0.5 h and 110 V for 1 h. The protein bands were transferred to polyvinylidene difluoride membranes using Trans-Blot Turbo Transfer System (Bio-Rad). Bovine serum albumin (5%) was added for 2 h to block the membranes. Thereafter, each membrane was further blocked using 5% bovine serum albumin for 1 h and then washed with Tris-buffered saline containing Tween (TBST). The primary antibodies were diluted as listed in Table 1. The tissues were incubated with each antibody at 4 °C for 16 h overnight. Then, the membranes were rewashed with TBST and incubated with the secondary antibodies. After incubating with the corresponding secondary antibodies at 28 °C for 1 h, the membranes were analyzed by enhanced chemiluminescence detection. The gray values of the immunoreactive protein bands were quantified using Image J (NIH, Bethesda, MD, USA).

### Immunofluorescence Staining

Frozen sections were used for immunofluorescence (IF) staining. Pancreas tissue was fixed in 4% paraformaldehyde overnight and dehydrated in 30% sucrose in 0.1 M PBS (Biosharp Life Sciences, China) at 4 °C. After embedding in optimal cutting temperature compound, the pancreas tissue was sliced into 10-μm-thick sections and mounted on slides. The sections were then blocked in 0.2% Triton X-100 (Sigma-Aldrich (Shanghai) Trading Co., Ltd.) for 10 min and permeabilized in Sea BLOCK Blocking Buffer (Thermo Fisher Scientific, USA) for 1 h. They were then incubated with primary antibodies against insulin (1:100, SAB) overnight at 4 °C, followed by incubation with secondary antibodies Alexa Fluor 488 (goat anti-rabbit, 1:500, Abcam, Cambridge, UK), Alexa Fluor 594 (goat anti-mouse, 1:500, Abcam, Cambridge, UK), or Alexa Fluor 405 (Goat anti-rabbit, 1:500, Abcam, Cambridge, UK) for 1 h at 37 °C. Different combinations of secondary antibodies were used to obtain optimal images. Finally, the tissue sections were covered by coverslips after washing them with 0.1 M PBS. Images were obtained by a fluorescence microscope (Olympus BX60 Darkfield DIC Metallurgical Microscope, Japan).

### Recording of Electrophysiological Activity in PINS

In order to obtain the discharge of the pancreatic intrinsic nervous system, the rats were anesthetized with isoflurane inhalation (2–5%) via a precision vaporizer (RWD Life Science Co., Ltd., Shenzhen, China). The depth of anesthesia was assessed by the absence of corneal and hind paw withdrawal reflexes. A laparotomy incision of approximately 3 cm was made in the skin just to the right of the abdomen midline. Dissection of the longitudinal muscle with adherent myenteric plexus has been dissected from the duodenum, leaving intact the connective tissue between the bowel and pancreas [37]. The pancreatic nerves run along the splenic artery and superior and inferior pancreatic arteries [38, 39]. The branch of the pancreatic intrinsic nerve was separated and connected to the positive electrode (PFA-Coated Platinum, A-M Systems, USA, 772000). The reference electrode was attached to the surrounding tissue. The experimental rats are placed in Faraday cages to shield them from electromagnetic interference signals. To avoid signal interference, only the electrodes that touch the nerves were exposed [40]. Spikes were recorded using a preamplifier (A-M Systems, Carlsborg, WA, USA; band-passing: 10–1000 Hz, sampling frequency: 20,000 Hz, and amplification: 1000-fold) and connected to a biosignal acquisition and analysis system (Microl 1401-3, CED, UK). The data were analyzed with Spike2 software.

### Acupuncture Intervention

EA refers to the application of a pulsating electrical current to acupuncture needles for acupoint stimulation [41]. The rats in the EA group received EA treatment on bilateral ST25 (Tianshu, located 5 mm lateral to the intersection between the upper 2/3rd and the lower 1/3rd in the line joining the xiphoid process and the upper border of the pubic symphysis) after gas anesthesia with isoflurane (2–5%). Meanwhile, the same anesthesia was administered to rats in the model group but without performing EA. For the EA group, two stainless steel acupuncture needles (Hwato, 20162270970, Suzhou, China) of 0.2 mm in diameter were inserted at a depth of 5 mm into the ST25 acupoint. EA at ST25 was conducted with the HANS-100A (Han Acuten, WQ1002F, Beijing, China) apparatus set to a current of 2 mA and a frequency of 2/15 Hz. All acupuncture procedures were performed by an experienced and licensed acupuncturist: 30 min a day for 6 days a week, 1 week a course, over four continuous courses of treatment.

### Data Analysis

Data from all the experiments are expressed as mean ± standard error values. Paired *t*-test was used for comparison before and after EA intervention, and an independent *t*-test was used for comparison between the two different groups. Multiple group comparisons were conducted using one-way ANOVA. All data analyses were performed using SPSS 22.0 software (IBM Corp., Armonk, NY, USA), and GraphPad Prism 8.0 (GraphPad Inc., La Holla, CA, USA) was used for data analysis. *p* < 0.05 was considered to indicate statistical significance.

## Results

### EA at ST25 Can Decrease Blood Glucose Level and Weight and Improve IVGTT

**Fig. 2:**
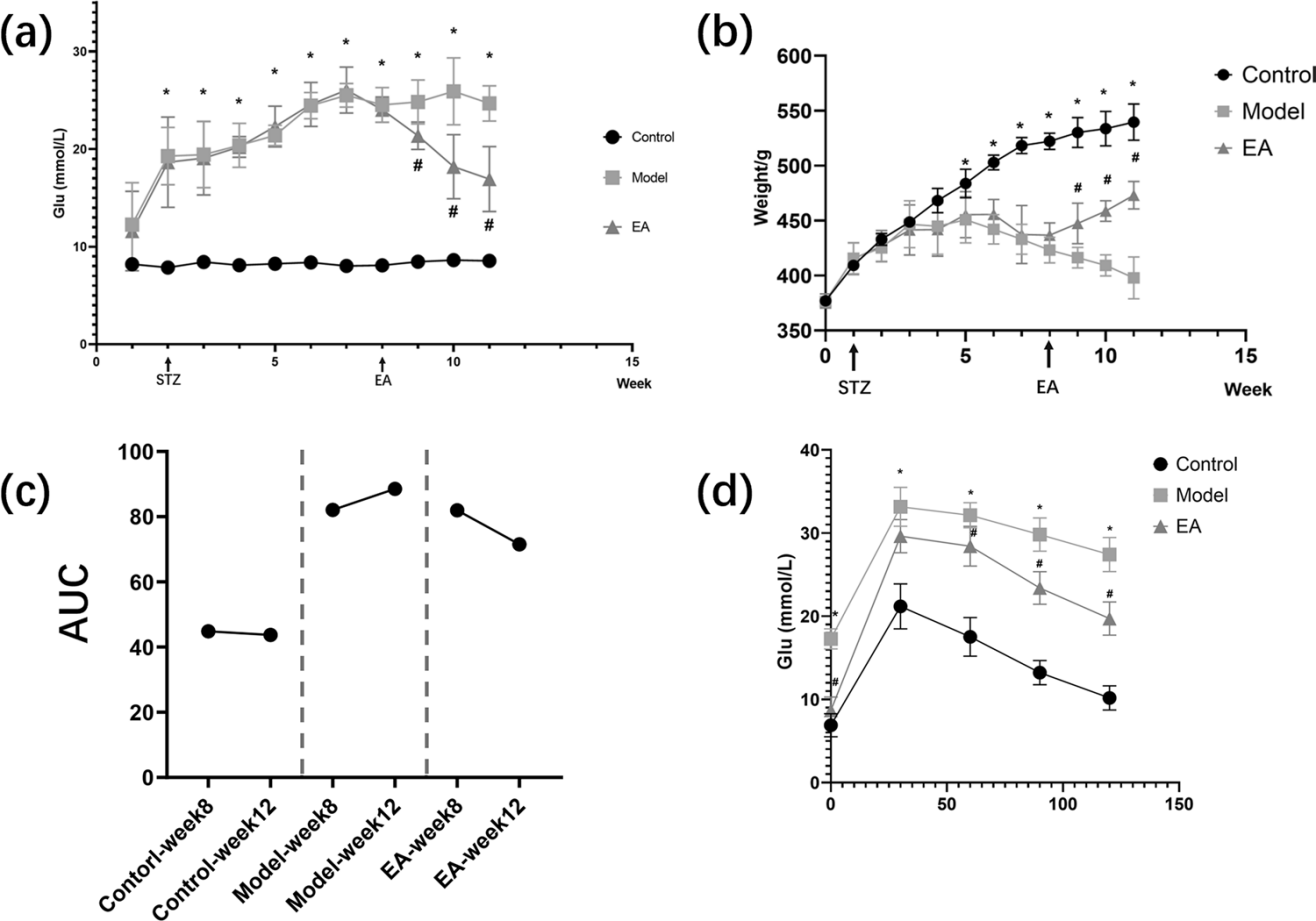
a Random blood glucose levels of rats during the experiment. b Weight of rats during the experiment. c Area under the curve (AUC) for the IVGTT test. d IVGTT test results after treatment. Results were expressed as mean ± SEM (n = 6). Data were analyzed by one-way ANOVA; paired t-test was used for comparison before and after EA intervention; *p < 0.05 vs. control, #p < 0.05 vs. model

The model group’s blood glucose level was higher than that of the control group (*P* < 0.05) from the second week and continued to increase, as shown in Fig. 2a. The model group’s weight was lower than the control group from the fifth week (*P* < 0.05), as shown in Fig. 2b. The hyperglycemia and the high body weight of the model group were maintained during the treatment, which lasted for 4 weeks. The blood glucose level and weight gain in the EA group decreased from the second week of treatment. These changes were statistically significant in comparison with those in the model group (*P* < 0.05). Moreover, these effects persisted for 3 weeks until the end of the experiment.

The glucose tolerance test was more sensitive than FPG alone in diagnosing T2DM. FPG or random blood glucose levels could not be used as a comprehensive measure of glycemic control. Intravenous glucose tolerance test (IVGTT) was performed at the end of week 8 (the last week before EA treatment) as well as week 12 (the last week of the experiment) to show the curative effect of EA. Additionally, the area under the curve (AUC) for the IVGTT test was computed as a measure of total glucose exposure. Figure 2c reveals that 4 weeks of EA improved glucose tolerance in rats. The AUC of the EA group was significantly lower than that of the model group (*P* < 0.05). For the EA group, the AUC after treatment was smaller than that before EA (*P* < 0.05, not labeled). Moreover, a small rise in the AUC in the model group was also observed, which suggests that the condition of the untreated rats continued to deteriorate. Furthermore, Fig. 2d shows the results of the IVGTT after treatment. The blood glucose level in the EA group decreased from 60 to 120 min and was lower than that in the model group (*P* < 0.05).

### EA at ST25 Improved Insulin Sensitivity and Controlled Inflammatory Status

**Fig. 3:**
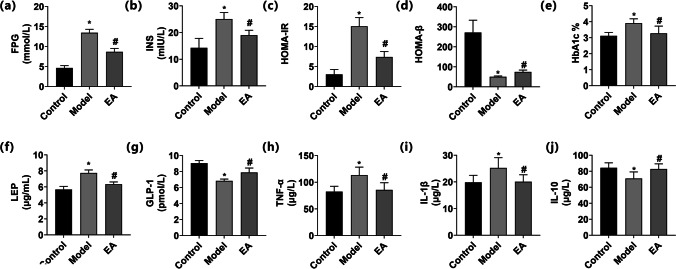
Comparison of β cell function and insulin resistance among groups. Fasting plasma glucose, FPG (**a**), fasting serum insulin, INS (**b**), HOMA-IR (**c**), HOMA-β (**d**), hemoglobin A1c, HbA1c (**e**), leptin, LEP (**f**), glucagon-like peptide-1, GLP-1 (**g**), tumor necrosis factor-α, TNF-α (**h**), IL-1β (**i**), and IL-10 (**j**). Results were expressed as mean ± SEM (n = 6). Data were analyzed by one-way ANOVA. *p < 0.05 vs. control, #p < 0.05 vs. T2DM

The maintenance of normal blood glucose levels is critical for the body to function properly. Markers of carbohydrate metabolism, such as glucose and insulin, are strongly associated with health problems in HFD-STZ rats. Data showing a comparison of *β* cell function and IR among groups can be found in Fig. 3. Insulin plays a key role in controlling blood glucose levels. The FPG and insulin of the model rats were significantly higher than that in the control rats (*P* < 0.01, Fig. 3a and b). EA at ST25 resulted in a decrease in FPG and insulin in comparison with the values in model rats (*P* < 0.01). Accordingly, HOMA-IR of the model rats was significantly higher than that in the control rats (*P* < 0.01, Fig. 3c), while EA at ST 25 led to a decrease in HOMA-IR when compared to model rats (*P* < 0.01). Remarkably, the HOMA-*β* of the model rats was much lower than in the control rats (*P* < 0.01, Fig. 3d). However, the EA group showed a higher HOMA-*β* than the model group (*P* < 0.01). Thus, the glucose metabolism of rats was normalized to a degree after EA but did not completely recover.

For decades, hemoglobin A1c (HbA1c) has remained the standard biomarker for glycemic control [42]. The HbA1c level of the model rats was higher than that in the control rats (*P* < 0.01, Fig. 3e). EA at ST25 decreased the HbA1c level in comparison with that in model rats (*P* = 0.114). Leptin, a hormone secreted from adipose tissue, plays a key role in energy balance and feeding behavior through neuronal regulation. Both leptin deficiency and leptin resistance are associated with the development of obesity [43]. The leptin level in the model group was higher than that in the control group (*P* < 0.01, Fig. 3f), while the leptin level in the treatment group was lower than that in the model group (*P* < 0.01). GLP-1 is a peptide hormone secreted from enteroendocrine L-cells into the hepatic portal circulation in response to ingestion of nutrients [44]. The GLP-1 level in the model group was lower than that in the control group (*P* < 0.01, Fig. 3g), while the GLP-1 level in the treatment group was higher than that in the model group (*P* < 0.01). T2DM is a polygenic disease with a low-grade inflammatory component. TNF-*α* is an inflammatory cytokine produced by various cells, including immune cells and epithelial cells [45]. Interleukin-1*β* (IL-1*β*) has been reported to contribute to *β* cell failure, and therapies targeting IL-1*β* have shown encouraging progress, albeit with diverse results in different clinical trials [46]. TNF-*α* and IL-1*β* of the model group were significantly higher than that of the control group (*P* < 0.05, Fig. 3h and i). EA at ST25 resulted in a decrease in TNF-*α* and IL-1*β* compared to model rats (*P* < 0.05). IL-10 is an anti-inflammatory cytokine that is known to suppress effector T cell responses and limit inflammation [47]. IL-10 level in the model group was lower than that in the control group (*P* < 0.05, Fig. 3j). The IL-10 level in the treatment group was higher than that in the model group (*P* < 0.05).

### Restoration of Islet Morphology Through Pancreatic Intrinsic Nervous System by EA

**Fig. 4:**
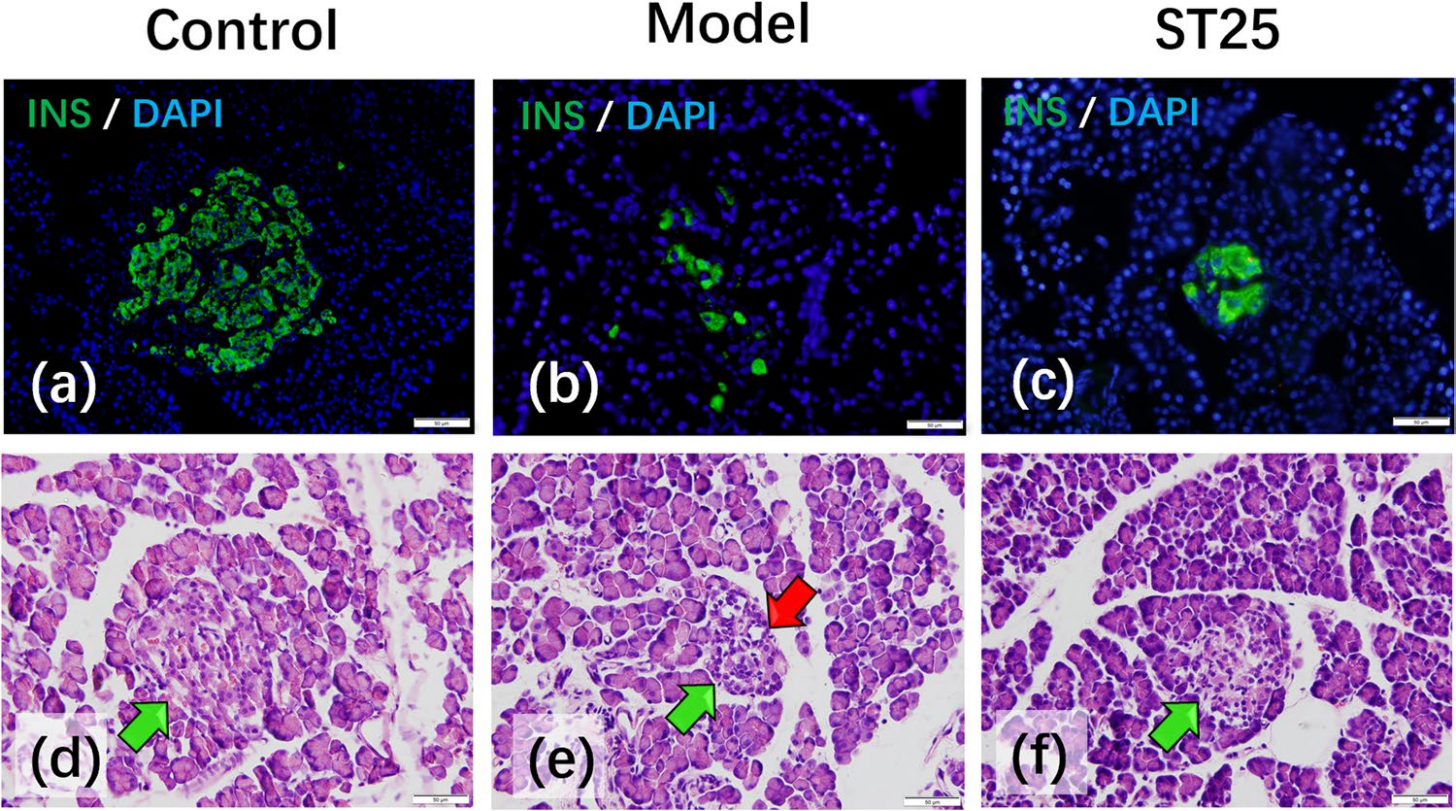
Representative HE and IF images of the pancreas. Green arrowheads show islets. Red arrowheads represent vacuoles. DAPI stained the nuclei (blue), and the green immunofluorescence represents the insulin. Islets were observed under a microscope (×400 magnification). Scale bar = 50 μm. The three groups share scale bars

Histological analysis was performed to observe islet morphology (Fig. 4). The pancreatic islet area of normal control rats revealed a normal architecture without any *β* cell damage. The morphology of islets showed a preserved round shape in the control group (Fig. 4a and d). In contrast, HFD-STZ-induced diabetic rats showed moderate damage and swelling of pancreatic *β* cells (Fig. 4b and e). Hyperglycemia led to marked changes in islet morphology [48]. The edges were irregular, the islet area was decreased, and the cells contained significantly more vacuoles. The islet shape was regular in the EA group (Fig. 4c and f). The morphology of islets in the ST25 group closely resembled that of the intact islets. EA improved the islet cell morphology by increasing the islet area, reducing vacuolation compared to those of the model group.

**Fig. 5:**
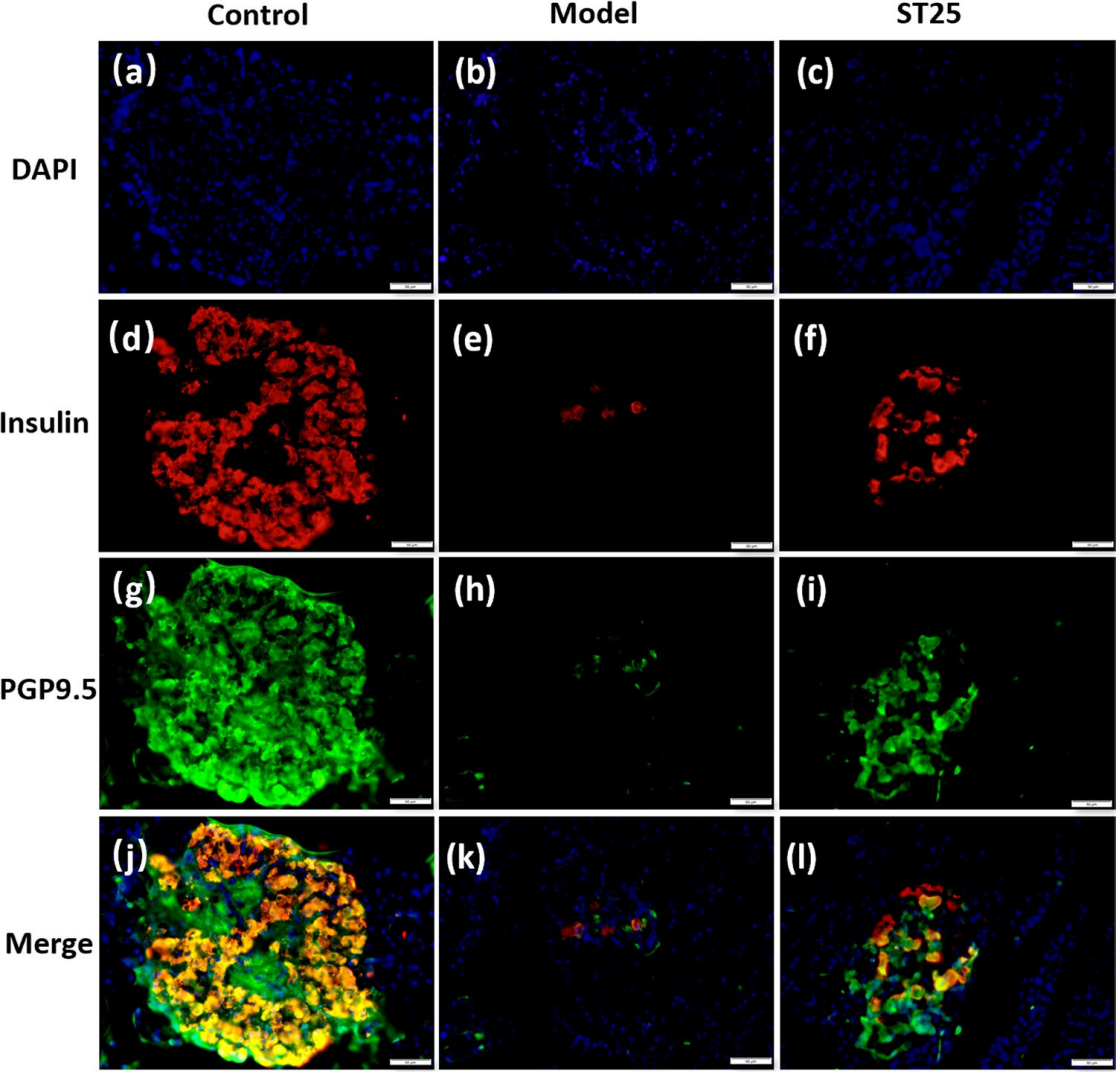
Representative IF images of the pancreas. DAPI stained the nuclei (blue), while the red immunofluorescence represents the insulin, and the green immunofluorescence represents the PGP9.5. The yellow color in the merged pictures indicates co-expression. Islets were observed under a microscope (×400 magnification). Scale bar = 50 μm. The three groups share scale bars

**Fig. 6:**
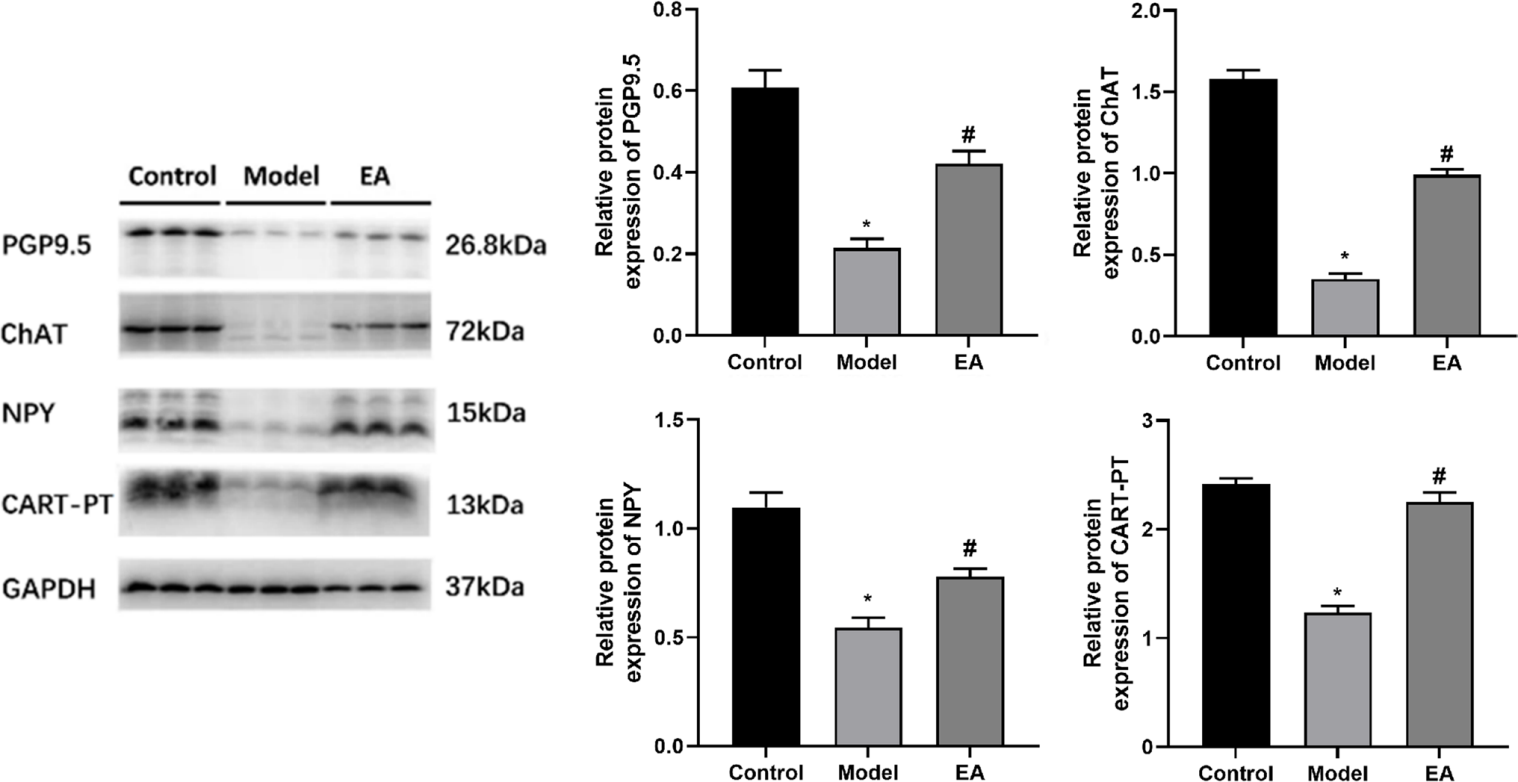
The effect of EA on pan-neuronal marker protein gene product 9.5 (PGP9.5), ChAT, NPY, and CART-PT expression. GAPDH was used as an internal reference protein, *p < 0.05 vs. control, #p < 0.05 vs. model

The pancreatic intrinsic nervous system was damaged during T2DM (Fig. 5h and k). The pan-neuronal marker protein gene product 9.5 (PGP9.5) was first examined by WB to explore the hypoglycemic effect of electroacupuncture at ST25 through neural regulation of pancreatic endocrine secretion. The expression of PGP9.5 in the pancreatic tissue of the model rats was significantly lower than that in the control rats (*P* < 0.05, quantification of PGP9.5 was checked by WB and is shown in Fig. 6). EA at ST25 resulted in an increase in PGP9.5 expression in comparison with model rats (*P* < 0.05). These findings were validated with the results of another experiment as described below (Fig. 6).

More research has been performed to identify specific neurotransmitters that can be modulated through EA at ST25. Additionally, the levels of ChAT, CGRP substance P (SP), NPY, and CART-PT expression decreased in the model group (*P* < 0.05), indicating PINS remodeling. The expression of these neurotransmitters improved after EA at ST25, as shown in Fig. 6.

### Pancreatic Endocrine Function Was Restored Through the TRPV1-(SP/CGRP)-β Cell Circuit

**Fig. 7:**
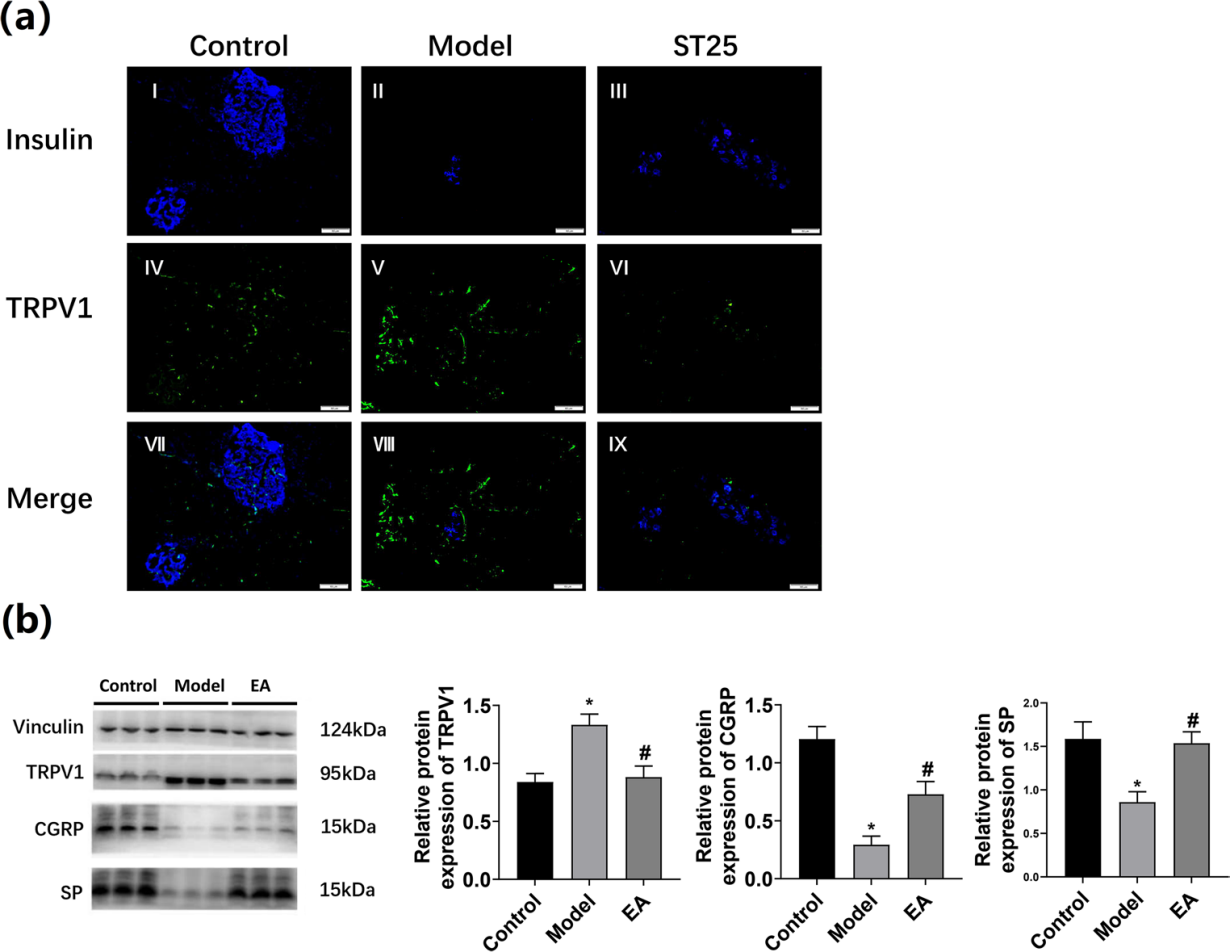
**a** Immunofluorescence staining and identification of TRPV1 (green) and insulin (blue) expression in the rat pancreas. Islets were observed under a microscope (×400 magnification); **b** The effect of EA on the expression of vanilloid 1 (TRPV1), CGRP, and SP. Vinculin was used as an internal reference protein. *p < 0.05 vs. control, #p < 0.05 vs. model

For further investigations, we also observed the expression of neural markers of sensory neurons has been observed, too. We focused on the expression of CGRP and SP since they can be regulated through transient receptor potential vanilloid 1(TRPV1) [49]. On the basis of the findings obtained with the observation of insulin in rat pancreas, we further evaluated the results of immunofluorescence staining and identified the expression of TRPV1 and insulin in rat pancreas to explore the neural regulation of pancreatic endocrine secretion through EA (Fig. 7a). In comparison with the model control group, the model group showed an increased fluorescently stained area of TRPV1 and a reduced area in the EA group. Quantification of TRPV1, CGRP, and SP in rat pancreas was performed by WB and shown in Fig. 7b.

### Electrophysiological Activity of PINS Before/After ST25 Acupuncture

**Fig. 8:**
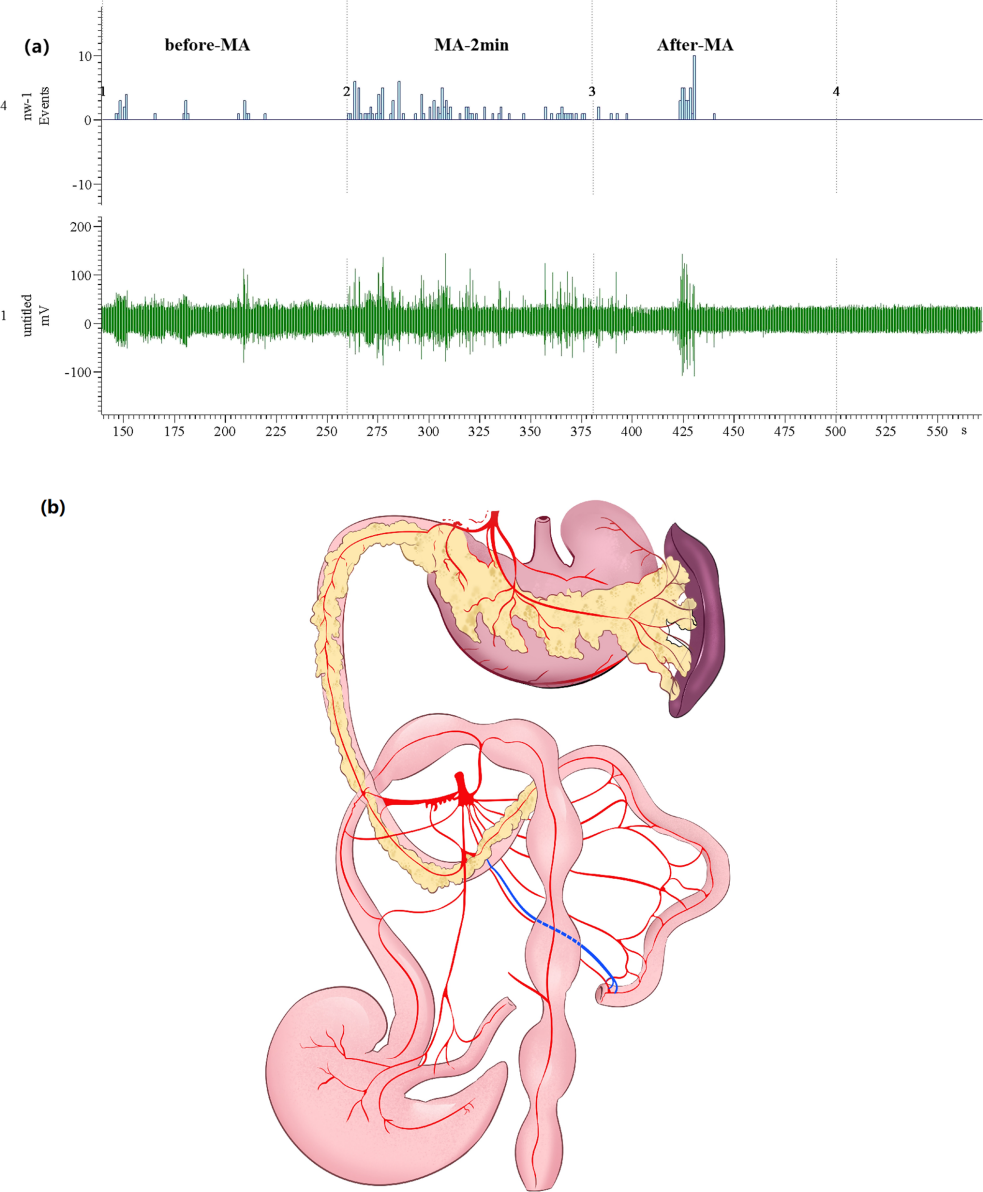
The effect of manual acupuncture at Tianshu (ST25) acupoint on the activity of PINS. **a** Waveform of PINS discharge by MA at ST25. **b** The anatomical location of PINS. The blue line represents PINS, which travels from the duodenum to the pancreas. The red lines show the blood vessels between the pancreas and duodenum (modified from Zoucas E 1996). MA, manual acupuncture; PINS, pancreatic intrinsic nervous system

In order to clarify the connections between ST25 and PINS, we then examined discharges of the PINS in normal rats. The activity of PINS during MA (2.71 ± 1.72 Hz) was significantly increased compared to the pre-MA frequency (0.32 ± 0.37 Hz, *P* < 0.05, Fig. 8a). The anatomical location of PINS is shown in Fig. 8b.

## Discussion

T2DM is characterized by IR combined with progressive pancreatic *β* cell failure [50], which can be moderated by acupuncture. The abnormal glycolysis process can affect lipid metabolism disorder, forming a vicious cycle [51], causing and aggravating oxidative stress, nerve damage, and other pathological changes [52]. In patients with diet-induced obesity, excess metabolites and lipid consumed in the diet result in high levels of circulating leptin, and the dysregulated leptin signaling maintains adipocyte hypertrophy and obesity [53]. Exogenous GLP-1 infusion increased plasma GLP-1 concentrations and caused a transient, but non-sustained, suppression of glucagon [54]. EA at ST25 can reduce HOMA-IR and increase HOMA-*β*. Specifically, after EA at ST25, the levels of fasting blood glucose, insulin, and glycated hemoglobin were all lower than those in the model group, while the level of GLP-1 increased. CART stimulates intestinal glucagon in a pathway independent of the known GLU Tag and STC-1 pathways [55] and increases Ca^2+^ signal transduction in the islet [56] in addition to altering islet *β* cells morphology [57]. Thus, the CART system may be an emerging therapeutic target for T2DM. In conclusion, as a GLP-1 regulator, CART can indirectly participate in the glucose metabolism of the pancreas. Additionally, EA at ST25 elevated the expression of leptin, as shown in Fig. 3, and leptin can also independently lower blood glucose levels [58].

EA had differential effects on metabolic markers in the HFD-STZ-induced rat model of T2DM. These effects may be explained neuroanatomically by variations in the segmental innervation of tissues at these locations [59, 60]. The acupuncture treatment reduced the HbA1c level significantly in comparison with that in the sham-acupuncture group [61]. The effectiveness of acupuncture in treating diseases related to IR had been reported, and acupuncture had an advantage over control groups in decreasing fasting blood glucose (FBG) and fasting insulin levels [62, 63]. Preventive acupuncture is beneficial for the control of STZ-induced hyperglycemia in rats [64]. EA intervention can significantly protect islet function and improve the FBG level in T2DM via regulation of thyroid hormone and phosphatidylinositol signaling [65].

As mentioned before, PINS constitute a complex information-processing center that includes various neurotransmitters and forms an endogenous neural network, which has an important influence on pancreatic endocrine function. PGP and multiple neurotransmitters have been shown to be upregulated by EA at ST25, suggesting adaptive changes in pancreatic-related nerves and transmitters. Those phenomena provided a neurophysiological basis for the onset of EA.

Chronic inflammation is closely related to pancreatic *β* cell damage [66]. EA at ST25 ameliorated the inflammatory state, contributing to the restoration of pancreatic *β* cell morphology (Fig. 4). Consistent with Ma’s research [16], the expression of NPY can explain the related experimental effects, including the anti-inflammatory effects. In addition, the changes in NPY expression over time showed the anti-inflammatory effect of EA at ST25, which can be observed in Fig. 3. In terms of inflammatory markers, the expression of TNF-*α* and IL-1*β* was decreased, while the expression of anti-inflammatory IL-10 was increased. The accumulation of pro-inflammatory factors can disturb the balance of apoptosis [67] and inhibit cell growth [68]. The anti-inflammatory effect of EA also indirectly protected *β* cells from apoptosis. Additionally, low-frequency electrical stimulation of the efferent vagal nerve fibers is thought to possess anti-inflammatory properties and can activate the “cholinergic anti-inflammatory reflex” [69]. ChAT is the most suitable factor for monitoring cholinergic neurons [70]. The increased expression of these neurotransmitters was consistent with the inflammatory activity, which verified the anti-inflammatory effect of EA at ST25.

Further explanation of our study focuses on the restoration of pancreatic endocrine function by EA at ST25 through the TRPV1 channel (SP/CGRP)-*β* cell circuit. The TRPV1 channel is highly expressed on sensory nerve fibers innervating the pancreas [71]. TRPV1 can be upregulated by high glucose levels [72], as observed in our study (Fig. 7b). It confirmed that TRPV1 was involved in the regulation of glucose. The pancreas receives sensory innervation, and its axon endings are sensitive to capsaicin and can release CGRP locally, which can induce diabetes [73].

In rodent models of T2DM, TRPV1 signal transduction is activated, and SP and CGRP release is increased chiefly. SP can inhibit glucose-induced insulin release and reduce glucose uptake, and thus improve insulin resistance [74]. However, we found that the expression of SP decreased in HFD-STZ-induced T2DM rats as the islets showed ill-conditioned patterns. This conclusion is consistent with Razavi’s assertion [75]. SP is one of the vital sources of pancreatic duct proliferation [76]. Pancreatic duct cells show the physiological characteristics of stem cells, which are differentiated into islet *β* cells [77]. Low levels of SP will lead to the proliferation of *β* cells and change glucose homeostasis [78]. Additionally, SP inhibits insulin secretion at low levels and promotes it at high levels [79]. In addition to TRPV1-expressing neurons, SP/CGRP also exists in islet cells and inhibits the release of insulin levels by *β* cells [80, 81]. Collectively, *β* cell activity was out of control as a result of deficient SP and CGRP.

Under physiological conditions, the functioning of TRPV1 will be affected by *β* cells. Chemical ablation of TRPV1 neurons can affect the function and quantity of islet *β* cells and improve glucose metabolism, indicating that *β* cells are vital targets of TRPV1 neurons [82]. The decrease in SP and CGRP can further activate the TRPV1 receptor through a positive feedback loop. However, there is a physiological limit to the expression level of TRPV1 receptors [32]. TRPV1 receptors degenerate after overstimulation, and although they remain at a high level, their sensitivity to stimulation decreases. SP/CGRP lacks the activation of the corresponding receptors, so it presents a low-level pathological state. Eventually, insulin will accumulate excessively, leading to the development of IR. This kind of TRPV1 channel (SP/CGRP)-*β* cell circuit balance disorder can lead to hyperinsulinemia, induce systemic IR, and disrupt glucose homeostasis.

In this study, we found that EA at ST25 could remarkably reduce IR and partially restore *β* cell function in T2DM rats. Further analysis showed that ST25 stimulation restores vital pancreatic functions regulated by PINS, regardless of pancreatic health, with transmitters such as NPY playing a critical role in this effect. Therefore, the significantly decreased HOMA-IR caused by stimulation at ST25 may be mediated via nervous innervation of the acupoint areas and imply the role of the PINS. WB and IF analyses showed that protein expression of PGP9.5 returned to nearly control levels after EA stimulation at ST25.

However, the islets of Langerhans make up only about 2% of the mass of the pancreas, so extrapolating the results from the whole pancreas to the endocrine pancreas is difficult. Although TRPV1 and CGRP/SP are of substantial significance in the treatment of T2DM [59], the neuroendocrine communication mechanism formed by TRPV1 and CGRP/SP is still unclear and needs further study.

## Conclusions

Current therapeutic strategies to manage hyperglycemia do not halt (or reverse) disease progression and may even cause undesirable adverse effects and comorbidities on their own [83], while treatment with insulin, sulfonylureas, and glinides may lead to weight gain [84]. EA at ST25 can reduce the body weight of T2DM rats and improve glucose metabolism. Using a high-fat-fed, STZ rat model that shows the metabolic characteristics of human T2DM, our study tested the hypothesis that EA at ST25 would repair the pancreas after STZ injury through neural regulation of the pancreatic intrinsic nervous system.
